# Neurodevelopment Outcome of Neonates Treated With Intraventricular Colistin for Ventriculitis Caused by Multiple Drug-Resistant Pathogens—A Case Series

**DOI:** 10.3389/fped.2020.582375

**Published:** 2021-01-20

**Authors:** Kashif Hussain, Muhammad Sohail Salat, Gul Ambreen, Javaid Iqbal

**Affiliations:** ^1^Department of Pharmacy, Aga Khan University Hospital, Karachi, Pakistan; ^2^Department of Pediatrics & Child Health, Aga Khan University, Karachi, Pakistan

**Keywords:** colistin, intraventricular antibiotics, neonates, multiple drugs resistant, ventriculitis

## Abstract

**Background:** Multiple-drug-resistant Gram-negative bacteria (MDR-GNB)-associated neonatal ventriculitis is a life-threatening complication that needs timely diagnosis and effective treatment with broad-spectrum antimicrobials in critical-care settings. Inadequate penetration of antibiotics through the blood–brain barrier also demands an intraventricular (IVT) route of administration. This study reports mortality and neurodevelopmental sequelae of neonates till 18 months of age, who received IVT-colistin for treating MDR-GNB associated ventriculitis.

**Methods:** In a case series of seven neonates with ventriculitis due to MDR-GNB at NICU of Aga Khan University Hospital, Pakistan, between June 2015 and 2018, we reviewed IVT-colistin therapy in critically ill neonates. Treatment outcomes were assessed based on clinical sign's resolution and MDR-GNB eradication in subsequent CSF cultures. Neurodevelopmental outcomes were evaluated at 18 months after discharge.

**Results:** The average birth weight was 1.38 kg (range: 1.02–1.5 kg), and the average gestational age was 30.7 weeks (ranged: 26–34 weeks). All neonates reported colistin-sensitive MDR-GNB in CSF, five with *Acinetobacter baumannii*, and polymicrobial CNS infection was found in two patients (one due to *Klebsiella pneumonia* and *A. baumannii* and one due *to K. pneumonia* and *Escherichia coli*). All neonates received IVT colistin and concomitant intravenous meropenem, and five of them also received intravenous colistin. One neonate died. At the 18-month assessment, only one neonate had cerebral palsy and hydrocephaly and 50% had seizure disorders.

**Conclusion:** Practicing intraventricular antibiotics in the neonatal population is challenging but may be used successfully, especially to overcome the limitation of poor penetration through the blood–brain barrier.

## Background

Central nervous system (CNS) infections are caused by several organisms, clinically presented as meningitis, ventriculitis, and brain abscess. The type of organisms involved and their susceptibility to available antimicrobials are the important factors in the clinical outcome and survival of these patients. It is crucial to timely recognize and manage these patients to reduce morbidity and mortality rates.

Nosocomial CNS infections are mostly a significant complication in patients undergoing neurosurgery (1), ranging from 0.3 to 6.5% in these patients (2–4). Its occurrence is about 8% in patients with external ventricular drain (EVD) (5, 6). In addition, one-third of the ventriculoperitoneal (VP) shunts implanted may lead to CNS infection (7). Neonatal CNS infections may occur concomitantly with the onset of Gram-negative pathogen-associated bacteremia (8–10). The overall neonatal mortality due to infections have reduced over the last few decades, but neonatal CNS infection-related morbidity remains almost unchanged (11, 12). Recent research has been focusing on improved diagnostic and preventive techniques and adjunctive therapies for improved neonatal outcomes (13).

The type of causative organisms of neonatal CNS infections depends on the gestational/postnatal age and facility setting (11, 14, 15). The treatment target for the nosocomial infections mostly involves multiple-drug-resistant (MDR) Gram-negative bacteria (GNB) such as *Acinetobacter baumannii* and *Klebsiella pneumoniae* (16). Studies have reported higher mortality ranges from 71.3 to 72.6% in patients with MDR-GNB-associated ventriculitis after neurosurgical procedures, mostly because of microbial resistance and inadequate antimicrobial penetration through the blood–brain barrier (BBB) (17, 18). Similarly, Chen et al. have reported up to 30% mortality rates in patients with carbapenem-resistant organism-associated CNS infections (19).

Fortunately, *A. baumannii* and *K. pneumoniae* are found susceptible to colistin, but limited penetration of colistin to cerebrospinal fluid (CSF) remains another challenge. These subtherapeutic levels require very high intravenous (IV) colistin doses. Consequently, the intrathecal (IT) or intraventricular (IVT) route is more appropriate to achieve the obligatory therapeutic colistin concentration to combat the MDR-GNB (20–24).

In adults and pediatric patients, neurological complications and sequelae of CNS infection have been studied. More severe illness, specific pathogens, earlier onset of paresis, and seizure have been reported as associated risk factors (25–27). Ventriculitis secondary to meningitis may occur in >20% of neonates (28). Survivors of neonatal meningitis and ventriculitis are at a much higher risk for neurodevelopmental impairments including motor function, hearing, vision, hydrocephalus, and seizure disorder.

Until today, very limited data is there about the usage of IVT/IT colistin in neonates, mostly in the form of case reports (29–31). That is not enough for the development of an exact guideline for IT/IVT colistin use in neonates for MDR-GNB-associated CNS infection. We encountered seven neonates in our neonatal intensive care unit (NICU) in 3 years, who had MDR-GNB-associated ventriculitis and treated with IVT colistin. Here, we are sharing the onset of CNS infection, treatment, and outcome of neonates in terms of survival and neurological complications.

## Method

### Study Design and Data Collection

In this retrospective case series, we reviewed the medical record of seven neonates at our 24-bedded NICU and the step-down unit of the Aga Khan University Hospital, Karachi, Pakistan, between June 2015 and June 2018. All the neonates included for the study were reported with the isolation of MDR-GNB from the CSF, developed ventriculitis during the NICU stay, and received colistin *via* the IVT route for >72 h.

Due to the high mortality rate with the emergence of MDR-GNB infections and the concern about poor CNS penetration of IV colistin, we offered adjunctive IVT colistin treatment to these neonates who had CNS infection. Infection control and preventive measures were strictly followed by the healthcare providers, such as contact isolation and hand hygiene.

For each case, data were collected regarding the demographic characteristics (gestational and postnatal age, sex, birth weight); primary and current diagnosis; the clinical and laboratory information about the infections; onset of CNS infection; hematological parameters (hemoglobin, neutrophil count, platelet counts, white blood cell (WBC) count, and C-reactive protein); presence of medical devices; concomitant antibiotic use along with susceptibility pattern, dose, and duration of IV; IVT colistin therapy; days to sterilized CSF; and IVT colistin-associated adverse effects and the outcome. After discharge, all surviving patients underwent a neurological examination performed by a pediatric neurologist, and the record was reviewed for the neurodevelopmental assessment reports, based on the broad classification of status at 18 months of age. It comprised of documentations of abnormal neurodevelopmental outcomes like motor dysfunction (cerebral palsy or monoplegia) and visual and hearing deficit with standardized criterion.

### Treatment Regimen of Intraventricular Colistin

Colistimethate sodium (CMS) is available in a vial containing 80 mg dry powder equivalent to 33.3 mg colistin base and further diluted in 2 ml of sterile preservative-free normal saline (NS). Colistin (base) was administered at a dose of 0.16–0.24 mg/kg as a single daily dose *via* the intraventricular route (21, 32, 33). All IVT doses were dispensed to nursing units in prefilled ready-to-administer form. The dose was administered through EVD by the neurosurgery team. Neonates were monitored for any hemodynamic changes during and after each dose (34).

### Operational Definition

Nosocomial infections were defined according to the standard definition by the Centers for Disease Control and Prevention (35). The IVT colistin-associated most common and life-threatening adverse effect is reversible chemical ventriculitis, which presents with sterile CSF cultures and signs of meningeal irritation, that is, altered mental state, fever, increased WBC, and lower glucose concentration in the CSF, and the presentation is like bacterial meningitis (21). MDRO was defined as an isolated pathogen non-susceptible to at least one agent of ≥3 antimicrobial categories (36). MDR-GNB-associated meningitis was defined as the presence of MDR-GNB in CSF cultures, and clinical presentation of neonatal meningitis is defined as the presence of signs like hyper or hypothermia, respiratory distress, hypotonia, lethargy or irritability, feed intolerance, apnea, lower heart rate and blood pressures, bulging anterior fontanel, seizures, and hypo or hyperglycemia (37). Choroid plexitis and inflammation of the ependymal lining of ventricles is called ventriculitis. Clinical symptoms of meningitis, failure to respond to appropriate antibiotic therapy, and signs of elevated intracranial pressure (ICP) suggest the diagnosis of ventriculitis. Further magnetic resonance imaging (MRI), lumbar puncture (LP), and fontanel tape aid in the diagnosis of ventriculitis (38, 39).

### Microbiology

For assessing the treatment response, the CSF of each neonate was collected and sent for chemical and microscopic analysis prior to IVT colistin administration. GNB were identified and checked for antibiotic susceptibility through the Vitek-2 compact system. Furthermore, colistin susceptibility of isolated GNB was confirmed by using the broth microdilution method. These isolated pathogens with minimum inhibitory concentration (MIC) of ≤2 mg/dl were documented as colistin-sensitive using Clinical Laboratory Standards Institute (CLSI) guidelines (40).

### Outcome Measures and Data Analysis

There were two primary outcomes of this study: (i) the cure of the patient fulfilling the following criteria: (a) MDR-GNB eradication in subsequent CSF cultures and (b) resolution of clinical signs of neonatal meningitis and with no further requirement of antimicrobial therapy; and (ii) neurodevelopmental outcomes till 18 months follow up after discharge. In the tables, we incorporated the demographic, clinical, treatment, and neurodevelopmental outcome details of each case.

## Results

During the study period, 3,264 neonates were admitted to our NICU. A total of 153 neonates received colistin therapy through any route. Of 153 neonates, 15 patients had culture-proven meningitis. Seven neonates developed ventriculitis and reported the isolation of colistin-sensitive MDR-GNB in CSF culture, thus treated with IVT colistin. Five of which were due to *A. baumannii*, and polymicrobial CNS infection was found in two patients (one of which was due to *K. pneumonia* and *A. baumannii* and one of which was due to *K. pneumonia* and *Escherichia coli*). All these critically ill neonates with MDR-GNB infections had received empirical antimicrobial therapy as per our institutional guidelines (41).

Neonatal characteristics, isolated MDR-GNB species, and treatment detail of each case are shown in Table 1. Out of seven neonates with MDR-GNB infections, five were male, the average birth weight was 1.38 kg (range: 1.02 to 1.5 kg), and the average gestational age was 30.7 weeks (range: 26 to 34 weeks). At the time of onset of ventriculitis, the average age of neonates was 26.9 days (range: 18 to 44 days). Five neonates had intraventricular hemorrhage (IVH), and one neonate had hypoxic–ischemic encephalopathy as associated diagnosis with ventriculitis. Two neonates had a history of pneumonia, and two had persistent pulmonary hypertension of the newborn (PPHN) before the onset of CNS infection. Four neonates had a VP shunt while three patients had no drainage system.

**Table 1:** Neonatal clinical characteristics and treatment outcome.

**Variables**	**Cases**						
	**1**	**2**	**3**	**4**	**5**	**6**	**7**
GA (weeks)	28	30	33	26	32	34	32
Gender	Female	Male	Male	Female	Male	Male	Male
BW (kg)	1.21	1.45	1.65	1.02	1.5	1.5	1.32
APGAR (at 1 & 5min)	7/8	9/9	1/2	1/3	6/9	3/5	5/9
MOD	Cesarean	Cesarean	Cesarean	delivered vaginally	delivered vaginally	delivered vaginally	Cesarean
Underlying illness	MAS, PPHN, IVH	RDS,IVH	HIE	RDS, VAP, IVH	Sepsis, IVH	sepsis, IVH and	TTN, PPHN
Age at the time of onset of CSF infection (days)	34	18	21	44	22	25	24
foreign body	VPS	VPS		VPS	-		VPS
Clinical manifestations of CSF infection onset	Hydrocephalus, Apnea	Hydrocephalus, fever	Hydrocephaly, fever, seizures and apnea	vomiting and feed intolerance, full fontanel, hydrocephalus	Hydrocephalus, bulging anterior fontanel, seizures	irritable, hydrocephalus, bulging anterior fontanel, feed intolerance, seizures	Hydrocephalus, vomiting, fever
**Laboratory investigations at onset of CSF infection**							
• WBC	• 2200	• 2700	• 1900	• 2500	• 2100	• 2300	• 2600
• Hb	• 11	• 7	• 10	• 9	• 11	• 11	• 10
• Plt	• 140000	• 75000	• 45000	• 70000	• 110000	• 130000	• 100000
• CRP	• 43	• 78	• 53	• 71	• 45	• 62	• 48
Isolated pathogens from CSF	*E. coli, KP*	*AB*	AB	*AB*	AB	AB	KP, AB
Antibiotic susceptibility	Colistin, tigecycline amikacin	Colistin, amikacin	Colistin	Colistin, tigecycline	Colistin and tigecycline	Colistin, tigecycline, and fosfomycin	Colistin, tigecycline
Antibiotics initially given before known susceptibility	Meropenem Vancomycin	Meropenem Vancomycin	Meropenem Vancomycin	cefotaxime amikacin, meropenem, colistin	cefotaxime amikacin, meropenem	cefotaxime amikacin, meropenem, vancomycin	Meropenem, ceftazidime and amikacin
Time between diagnosis of CSF infection and IVT colistin therapy (days)	4	3	7	12	4	4	5
Duration of IVT colistin (days)	7	7	5^*^	8	7	3^*^	8
Mean IVT colistin doses (mg/kg/day)	0.21	0.2	0.21	0.19	0.23	0.21	0.2
Duration of IV colistin (days)	14	14	15	21	14	-	-
Dose of IV colistin (mg/kg/day)	5	4.8	5	4.8	5	5	5
Duration of concurrent intravenous antibiotic therapy	Meropenem-14 amikacin 7	Meropenem-14 amikacin-3	Meropenem-14	Meropenem-14	Meropenem-10	Meropenem-14 amikacin-3	Meropenem-7
Blood culture	-	AB	AB	AB	AB	-	KP
**Outcomes**							
• CSF infection	Cured	Cured	Cured	Cured	Cured	Not Cured till death	Cured
• Patient	Discharged	Discharged	Discharged	Discharged	Discharged	Died	Discharged
Development of IVT colistin related adverse effects	No	No	Yes	No	No	No	No
Days to sterilize CSF	3	2	8	4	5	Not achieved	4

*GA, gestational age; BW, birth weight; MOD, mode of delivery; CSF, cerebrospinal fluid; CRP, C-reactive protein (mg/L); IVT, intraventricular; IV, intravenous; WBC, white blood cells count (cells/mm^3^); Hb, hemoglobin (g/dl); Plt, platelets count (cells/mm^3^); IVH, intraventricular hemorrhage; PPHN, persistent pulmonary hypertension of the newborn; MAS, meconium aspiration syndrome; RDS, respiratory distress syndrome; HIE, hypoxic ischemic encephalopathy; VAP, ventilator-associated pneumonia; TTN, transient tachypnea of the newborn; E.coli, Escherichia coli; KP, klebsiella pneumonia; AB, acinetobacter baumannii, VPS, ventriculoperitoneal shunt*;

* *doses given on alternate days*.

Based on the previous culture reports, susceptibility pattern, and clinical grounds, to treat the ongoing infections neonates were given other IV antibiotics. Three neonates were treated with cefotaxime and amikacin initially, then switched to meropenem, either alone or in combination with colistin or vancomycin. Three neonates were treated with meropenem plus vancomycin. One neonate received ceftazidime and amikacin after discontinuation of meropenem. Four neonates developed concomitant pneumonia (three with *A. baumannii* and one with *K. pneumoniae*). The blood cultures of five neonates were also positive with MDR-GNB (four with *A. baumannii* and one with *K. pneumoniae*).

From all the cases, five neonates were treated with concomitant IV colistin in the dose of 5 mg/kg/day in daily divided doses for an average duration of 15.6 days (range: 14 to 21 days), whereas all of seven neonates received concomitant meropenem and three of them were given amikacin. The average duration between the diagnosis of CSF infection and IVT colistin therapy was 5.6 days (range: 3 to 12 days), and the average period to attain sterile CSF among six survived neonates was 3.72 days (range: 2 to 8 days). Among all, one neonate died during the treatment, due to *A. baumannii*. Till the last examination, CSF was unsterile in this patient and had received three doses of IVT colistin on alternate days.

All six survived neonates who achieved the sterile CSF phase were cured without relapse after completion of the treatment course. On average, neonates received IVT colistin for 7.4 days (range: 7 to 10 days). Intraventricular colistin-associated adverse effect was developed in one neonate and thus received IVT colistin on alternate days.

Table 2 shows the results of the neurodevelopment assessment of survived infants at 18 months of age. One of the neonates who followed in the neurology clinic had cerebral palsy (CP), seizure disorder (on anticonvulsant therapy), and impaired hearing and vision. One neonate had hydrocephalus, epilepsy, and developed retinopathy of prematurity (ROP). Both cases were on follow-up with a neurologist. From the remaining four, one baby received ROP treatment and one was on antiepileptic; otherwise, these four babies were found normal at the 18-month assessment.

**Table 2:** Neurodevelopment assessment of survived children with ventriculitis at 18 months of age.

**Parameters**	**Case**					
	**1**	**2**	**3**	**4**	**5**	**7**
Motor dysfunction (cerebral palsy) (yes/No)	No	No	No	Yes	No	No
Hearing defect (Normal/mild/moderate/severe)	Normal	Normal	Normal	Moderate	Normal	Normal
Visual deficits (impaired/normal/ROP)	ROP	Normal	Normal	Impaired	ROP	Normal
Hydrocephaly /VP Shunt(Yes/No)	No	No	No	Yes	Yes	No
Seizure Disorder (Yes/No)	No	No	yes	Yes	yes	No

*ROP, retinopathy od prematurity*.

## Discussion

Ventriculitis secondary to meningitis is more common in neonates (42) and may occur in >20% of neonates with meningitis (28). Moreover, significantly higher morbidity and mortality due to MDR-GNB-associated meningitis and ventriculitis need extra precautionary measures and further therapeutic considerations to reduce the consequences of such life-threatening complications (17–19). Increased pathogen resistance to most of the antimicrobial agents and their poor CNS penetration are the contributing factors. Therefore, several studies have chosen the IVT/IT route of administration to directly deliver the antibiotic, to achieve the desired therapeutic steady state to fight against these pathogens (20, 21, 23, 43). Though meropenem is used empirically for nosocomial MDR-GNB-associated meningitis and ventriculitis (44, 45), unfortunately, it is becoming progressively ineffective due to rapidly increased resistance (more than 40%) among these pathogens (46). Fortunately, these MDR strains remain sensitive to polymyxins, an old class of antimicrobial agents from the 1950s (41). However, due to the polycationic structure and the higher molecular weight polymyxins have a poor capacity to cross the BBB when administered through the IV route (47). Consequently, IV colistin (polymyxin E) remains unable to achieve therapeutic CSF concentration to fight against deadly MDR-GNB (48).

For treating IVH and posthemorrhagic hydrocephalus in neonates along with the pharmacological approach, other modalities, such as EVD, serial lumbar or ventricular taps, and ventriculoperitoneal (VP) shunts, are used (49–51). In addition, for administering IVT/IT medications special tools are needed, such as EVD, but these foreign bodies serve as a potential infection source (52). In adult studies, the IT route of administration is used by a repeated lumbar puncture on daily basis; thus, removal of lumbar drain and EVD is preferred to prevent foreign bodies' systemic involvement (20). However, the IT route of administration is comparatively not feasible and difficult in neonates; therefore, IVT is preferred (53, 54).

The Infectious Diseases Society of America (IDSA) guidelines recommend using IV and IT/IVT polymyxins (polymyxin B and colistin) with meropenem in carbapenem-resistance strain-associated infections (52). The use of colistin through IVT/IT routes is advocated in adults' clinical practices, but neonatal studies are very limited (21). Karaiskos et al. reviewed IVT/IT colistin usage in multidrug-resistant gram-negative strain-associated meningitis and ventriculitis (21), involving 81 patients (71 adult and 10 children/neonates). The clinical and microbiological outcomes were achieved in 89% of patients. In addition, nine cases (11%) experienced an adverse effect of chemical ventriculitis. In our study, only one patient experienced this adverse effect. A comparatively shorter duration of IVT antibiotic use might explain this difference. The CSF cultures of our patients were persistently sterile, but chances of reinfection need to be considered. In another review, Katragkou et al. discussed the treatment of MDR *A. baumannii*-associated CNS infection by using IVT/IT colistin alone or as an adjunctive to systemic antimicrobials (55). In our study, neonatal CSF became sterile within an average of 3.72 days (range 2–8 days), which is similar to earlier literature (56, 57). Previous adult and pediatric studies have concluded that the use of IT/IVT colistin was found safe and effective (56, 57).

Aminoglycosides, tigecycline, rifampin, and sulbactam have also been used effectively as alternative therapeutic options (21). However, in our study meropenem was the only antibiotic used in all the seven patients concomitant with IVT colistin and IV colistin was used in 5/7 patients, which could be justified due to the presence of blood and pulmonary infections. In addition, combination therapy with colistin, meropenem, and ampicillin-sulbactam has been reported to reduce the mortality rate (21). Colistin resistance among *A. baumannii* and *K. pneumonia* are increasingly reported and a case report from South Africa identified the presence of colistin-resistant *A baumannii*-associated ventriculitis in a neonate (56). Another case report by Mehar *et al*. reported septicemia with meningitis due to carbapenem-resistant (susceptible to colistin) *A. baumannii* in a preterm neonate. The infant was successfully treated with IVT colistin and IV netilmicin (58). MDR-GNB-associated neonatal ventriculitis and meningitis is a challenge to manage using conventional IV antibiotics and found inadequate to eradicate these life-threatening pathogens. Fortunately, all the seven cases in our study had colistin-susceptible MDR-GNB-associated CNS infection and the MDR strain did not acquire resistance against colistin in any patient which resulted in 86% success with IVT colistin treatment.

A neonatal study for evaluating the neurodevelopmental defects had reported that from the neonates who experienced bacterial CNS infection and survived, 67% developed neurologic sequelae (59). In our study, one neonate died with uncontrolled seizures, and from survivors, only one neonate who had initial seizures presented with unfavorable neurological effects at 18 months. From six survived neonates, three developed seizures. These findings are also supported by a previous study, which shared that the presence of initial seizure is a significant predictor of unfavorable outcome (60). To the best of our knowledge, no study has been conducted to evaluate the neurodevelopmental effects at 18 months of age, in preterm neonates, who received IVT antibiotics for treating MDR-GNB-associated ventriculitis.

The increasing prevalence of MDRO causing neonatal sepsis including meningitis and ventriculitis in some middle- and low-income countries is a matter of great concern (41, 56). Also, the implementation of infection control and prevention strategies is a global challenge in these regions to control the spread of MDR-GNB. Furthermore, in our region with limited advanced NICU settings, the transfer of ill neonates from other facilities is an additional factor in the spread of MDR pathogens. Implementation of more firm screening measures may help to prevent the spread by detecting colonized patients earlier (61). Fortunately, in our NICU patient cohorting is done to keep the externally transferred and infected infants in the isolation room provided negative pressure. Our study had limitations of a single-centered retrospective case series study design, the association of confounders, and a small sample size.

## Conclusion

Multidrug-resistant pathogen-associated meningitis and ventriculitis are evolving in neonates, and currently available antimicrobials have deprived outcomes in CNS infection due to poor penetration through the blood–brain barrier. However, practicing intraventricular antibiotics in the neonatal population is challenging but may be used successfully. The efficient treatment approach with suitable antibiotic usage through an appropriate route of administration may enhance survival and improve neurodevelopmental outcomes. The spread of multidrug-resistant pathogens can be reduced through implementing and continuing practicing simple infection control and prevention measures. Further prospective, controlled studies are warranted.

## Data Availability Statement

The raw data supporting the conclusions of this article will be made available by the authors, without undue reservation.

## Ethics Statement

The studies involving human participants were reviewed and approved by Ethical Review Committee, Aga Khan University Karachi, Pakistan. Written informed consent to participate in this study was waived as the data was collected retroactively.

## Author Contributions

GA, MS, and KH conceptualized the design. GA and JI carried out data collection. KH and GA contributed to pharmacy data retrieval. GA wrote the draft. MS, KH, and JI carried out the analysis. MS supervised the study and critically revised the subsequent drafts. All authors contributed to the final manuscript.

## Conflict of Interest

The authors declare that the research was conducted in the absence of any commercial or financial relationships that could be construed as a potential conflict of interest.
